# Direct LiF imaging diagnostics on refractive X-ray focusing at the EuXFEL High Energy Density instrument

**DOI:** 10.1107/S1600577522006245

**Published:** 2023-01-01

**Authors:** Sergey Makarov, Mikako Makita, Motoaki Nakatsutsumi, Tatiana Pikuz, Norimasa Ozaki, Thomas R. Preston, Karen Appel, Zuzana Konopkova, Valerio Cerantola, Erik Brambrink, Jan-Patrick Schwinkendorf, Istvan Mohacsi, Tomas Burian, Jaromir Chalupsky, Vera Hajkova, Libor Juha, Vojtech Vozda, Bob Nagler, Ulf Zastrau, Sergey Pikuz

**Affiliations:** a Joint Institute for High Temperatures Russian Academy of Sciences, Izhorskaya St 13, Bd 2, Moscow 125412, Russian Federation; b European XFEL, Holzkoppel 4, 22869 Hamburg, Germany; c Institute for Open and Transdisciplinary Research Initiatives, Osaka University, 2-6 Yamadaoka, Osaka 565-0871, Japan; d Graduate School of Engineering, Osaka University, Suita, Osaka 565-0871, Japan; e Photon Pioneers Center, Osaka University, Suita, Osaka 565-0871, Japan; f Department of Earth and Environmental Sciences, Università degli Studi di Milano-Bicocca, Piazza della Scienza 4, 20126 Milan, Italy; g Department of Radiation and Chemical Physics, Institute of Physics, Czech Academy of Sciences, Na Slovance 2, 182 21 Prague 8, Czech Republic; h Plasma Physics Department, Institute of Plasma Physics, Czech Academy of Sciences, Za Slovankou 3, 182 00 Prague 8, Czech Republic; i SLAC National Accelerator Laboratory, 2575 Sand Hill Road, Menlo Park, CA 94025, USA; RIKEN SPring-8 Center, Japan

**Keywords:** X-ray free-electron lasers, X-ray focusing, X-ray beam characterization, compound refractive lenses, focusing system, lithium fluoride (LiF) detector

## Abstract

The application of fluorescent crystal media in wide-range X-ray detectors provides an opportunity to directly image the spatial distribution of ultra-intense X-ray beams including investigation of the focal spot of free-electron lasers. Here the capabilities of the micro- and nano-focusing X-ray refractive optics available at the High Energy Density instrument of the European XFEL are reported, as measured *in situ* by means of a LiF fluorescent detector placed into and around the beam caustic. The intensity distribution of the beam focused down to several hundred nanometers was imaged at 9 keV photon energy. A deviation from the parabolic surface in a stack of nanofocusing Be compound refractive lenses (CRLs) was found to affect the resulting intensity distribution within the beam. Comparison of experimental patterns in the far field with patterns calculated for different CRL lens imperfections allowed the overall inhomogeneity in the CRL stack to be estimated. The precise determination of the focal spot size and shape on a sub-micrometer level is essential for a number of high energy density studies requiring either a pin-size backlighting spot or extreme intensities for X-ray heating.

## Abstract 2

The focusing capabilities of the X-ray free-electron laser beam at the High Energy Density instrument at the European XFEL are imaged and studied by means of high-dynamic-range fluorescent LiF media placed directly into the beam caustic.

## 1. Introduction

The recent development of X-ray free-electron laser (FEL) technology opens the door for experimental physics of high-energy photon–matter interactions and great opportunities for advanced technology in material processing. State-of-the-art FEL facilities have recently been commissioned: LCLS (USA, 2009), SACLA (Japan, 2011), PAL-XFEL (Republic of Korea, 2016), SwissFEL (Switzerland, 2016), EuXFEL (Germany, 2017). The radiation provided by XFELs possesses most of the important features of lasing, such as coherency, narrow bandwidth, and close to diffraction-limited angular divergence. With up to a few mJ pulse energy, tight focusing and a few femtosecond pulse duration, radiation fields with extremely high intensity up to 10^20^ W cm^−2^ are now within reach. A wide range of possible parameters for XFEL beams (wavelength, photon flux, energy) makes it possible to study various processes in matter that occur under the influence of X-rays. In addition to that, focused beams of ultrahigh intensity can be used to develop new technological applications for nanoscale precise material processing with X-rays. Precise knowledge of the X-ray intensity profile at the interaction area is apparently crucial for any FEL experiment.

Since XFELs began to operate, many operational and autonomous methods have traditionally been used to monitor the wave properties of the beam. These include *out of focus* diagnostics (diagnostics performed away from the beam focus) such as Shack–Hartmann sensors (Keitel *et al.*, 2016 ▸), Young’s experiment with two slits (Vartanyants *et al.*, 2011 ▸), crystal spectrometry (Boesenberg *et al.*, 2017 ▸), speckle tracking (Berujon *et al.*, 2015 ▸), ptychography (Schropp *et al.*, 2013 ▸) and various grating-based methods (Makita *et al.*, 2020 ▸; Daurer *et al.*, 2021 ▸; Rutishauser *et al.*, 2012 ▸; Schneider *et al.*, 2018 ▸; Liu *et al.*, 2018 ▸); and, on the other hand, the traditionally practiced *in focus* methods (diagnostics at the focusing area) such as ablation imprinting (Chalupsky *et al.*, 2011 ▸, 2015 ▸) and the widely used knife-edge scanning approach (Yumoto *et al.*, 2013 ▸). Each of these methods has its own advantages and disadvantages.

In this work we applied imaging diagnostics based on *in situ* measurement of the intensity distribution by means of a lithium fluoride (LiF) crystal detector placed directly in the part of the beam under interest. The performance of this method in terms of characterizing the focusability and spatial structure of the XFEL beams has been demonstrated by Pikuz *et al.* (2015 ▸, 2018 ▸), where the possibility of recording the cross section of the intensity profile of the XFEL beam with high spatial resolution (∼1 µm), very large dynamic range (no less than 10^6^) and within a field of view larger than a few mm^2^ at a photon energy of 10 keV was shown. The combination of the significantly advantageous characteristics of the LiF detector represents a unique feature compared with conventional detectors such as image plates and CCDs. Makarov *et al.* (2020 ▸) observed the 2D intensity distribution of a diffraction pattern created by the PETRA-III X-ray beam with circular aperture up to the 16th order maximum, which required both a spatial resolution in the sub-micrometer range and a dynamic range of ∼10^7^. The micrometer-size resolution of the LiF detector allowed the characterization of the SACLA XFEL source and determination of the spatial and coherent properties of the beam by applying the high-resolution Fresnel diffraction method developed by Ruiz-Lopez *et al.* (2017 ▸). In this way, LiF as an *in situ* imaging detector is a very convenient instrument for acquiring intensity distribution patterns along the caustics of XFEL beams in any configuration: free-propagated direct beam or beam transformed by the X-ray focusing system. Thus, the focusing properties of the Kirk­patrick–Baez mirrors installed at BL3 of SACLA XFEL have been studied (Pikuz *et al.*, 2015 ▸).

At present, in experiments with XFELs, refractive focusing systems are used (Hagemann *et al.*, 2021 ▸; Maeda *et al.*, 2020 ▸; Seiboth *et al.*, 2018 ▸; Schropp *et al.*, 2015 ▸). Our work was done within the framework of the multi-approach experiment on the complex characterization of the refractive focusing system installed at the High Energy Density (HED) instrument at the EuXFEL (Schenefeld, Germany). Details of the whole campaign performed with the application of various diagnostics methods, including those mentioned above, is the subject of a special publication and will be described elsewhere. Here we report on imaging of the hard X-ray focus by means of *in situ* acquisition of XFEL patterns with a LiF detector and on the results of simulations that support our initial experimental observations.

## 2. Experimental method

**Figure 1:**
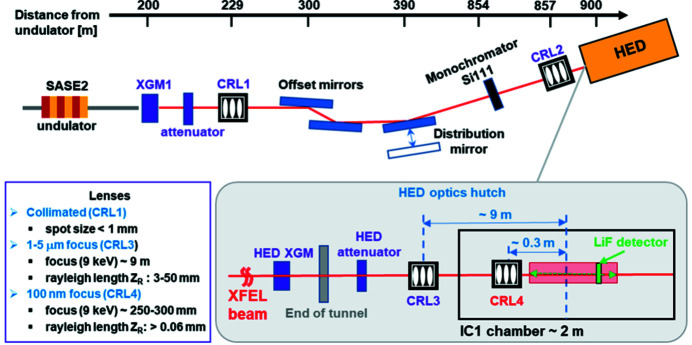
Schematic view of the X-ray beam transport from the SASE2 undulator to the HED experimental hutch, and parameters of CRL units 1, 3 and 4 (XGM – X-ray gas monitor for single-shot pulse energy measurements and average beam position monitoring; HED – High Energy Density instrument).

The experiment was carried out at the HED instrument, which is located at the SASE2 undulator. The X-ray beam transport from the undulator up to the HED experimental hutch is schematically shown in Fig. 1 ▸ [a detailed description of the X-ray transport optics is given by Zastrau *et al.* (2021 ▸)]. The X-ray focusing system of the HED instrument is based entirely on the use of a beryllium compound refractive lens (CRL) with parabolic surfaces of individual refractive lenses. The CRL is chromatic, and thus requires different lens configurations for different photon energies.

Four CRL lens units (1–4) can be used to focus the beam at different positions along the HED beamline (Zastrau *et al.*, 2021 ▸; Schneidmiller & Yurkov, 2011 ▸). In our experiment, the SASE2 undulator was tuned to deliver short X-ray pulses of ∼40 fs duration and peak pulse energy of ∼2 mJ at a photon energy of 9 keV. Beam characterization and quality assessments were performed for the beam focused using the third lens unit (CRL3) to a focal spot of several micrometers in waist, and for the beam focused using the fourth lens unit (CRL4) to a focal spot of sub-micrometer size. In both cases the focusing was provided in combination with the most upstream lens unit (CRL1) in the tunnel. The lens unit CRL1 is the most upstream focusing element on the beam transport line and can be used for collimation, direct focusing and intermediate focusing of the XFEL beam to CRL2 and CRL3. Table 1 ▸ shows the main parameters of the lenses used in the experiment. The diffraction-limited size of the beam in the focus, *d* = λ/NA, where λ is the wavelength and NA is the numerical aperture of the CRL, was ∼1.5 µm for CRL3 and ∼200 nm for CRL4 at 9 keV photon energy.

To study the focusing properties of lenses, it is necessary to know the intensity distribution within the beam not only at the focal plane but also at a distance along the laser propagation axis. The 3D spatial profile of the focused beam is bound by the surface of the caustic. The caustic of a Gaussian beam after focusing by an ideal optical system represents a hyperbolic surface. The angle between the asymptotes of the hyperbola defines the divergence of the beam. The position and the diameter of the waist define the position and the size of the focus. The distribution of radiation in the cross section of the caustic is well approximated by the Gaussian function. Following the theory of focused Gaussian beams, the spot diameter 2*r*_*z*_ (FWHM of the beam intensity on-axis) at distance *z* from the focal plane can be calculated using the equation (Self, 1983 ▸)

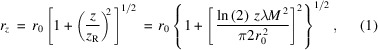

where *r*_0_ is the spot radius of the beam at the focal plane, *z*_R_ is the Rayleigh range, λ is the wavelength of the photon and *M* ^2^ is the beam quality factor.

To reveal the caustic of a focused beam, we used a LiF detector. The formation of images in a LiF detector crystal is based on the ability to create stable color centers (CCs) in the crystal under direct irradiation by photons with energy greater than 14 eV, whose absorption and fluorescence spectra belong to the optical range (Baldacchini *et al.*, 2005 ▸). This allows LiF to be used as a detector in which the image is encoded according to the density of the color centers in the crystal. Deep propagation of the X-ray beam into the LiF causes generation of CCs in the volume of the LiF crystal and allows the 3D structure of the beam to be visualized, for example for precise determination of the best focal position (Pikuz *et al.*, 2015 ▸). It should also be noted that the spatial resolution of the LiF detector depends on the photon energy. The reason for this is the influence of the secondary electron cascade generated in LiF by incident X-ray photons. In the work by Grum-Grzhimailo *et al.* (2017 ▸), it was theoretically predicted that the radius of the electron cloud for hard X-ray photons can reach several hundred nanometers.

In our experiment, we used circular LiF crystals of 20 mm diameter and 2 mm thickness mounted on an *XYZ* motorized stage. By moving a crystal along the X-ray beam (*Z*-axis), the intensity distribution in the cross section of the beam was recorded in sequences of planes *XY* near the focus over a *Z*-range of ∼300 mm, with a step size from 0.2 mm up to 2 mm (Fig. 1 ▸). The photoluminescent (PL) signal of the exposed LiF crystals was read out using a laser scanning confocal microscope (Carl Zeiss LSM700). To obtain a 2D image of the beam in values of intensity, the PL signal was recalculated by means of an algorithm developed by Bonfigli *et al.* (2021 ▸). It is known that the PL response of the LiF crystal depends only on the amount of absorbed energy in the crystal and does not depend on the energy of incident photons. In our calculation we applied the PL response function defined in recent works (Mabey *et al.*, 2019 ▸; Makarov *et al.*, 2020 ▸). This function was determined in a wide enough range of absorbed energies to be applicable for our experimental conditions.

## 3. Experimental results and discussion

### 3.1. Focusing properties of lens unit CRL1

**Figure 2:**
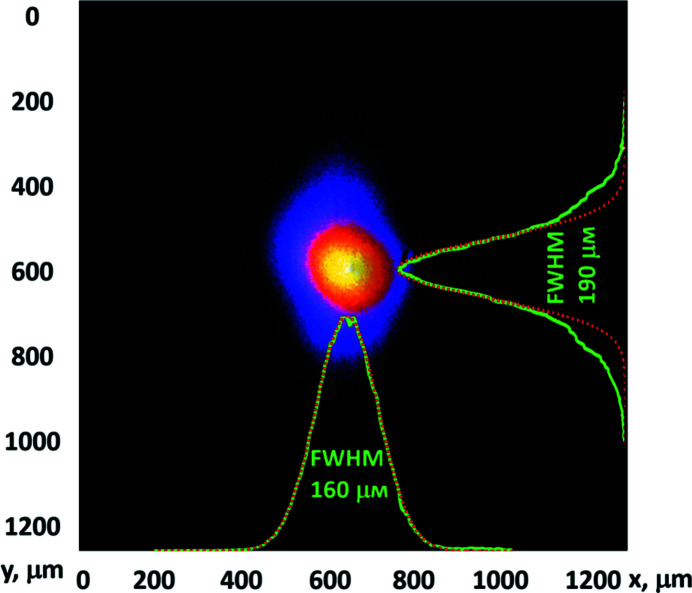
Image of the XFEL beam directly focused by the CRL1 lens measured by means of a LiF detector at IC1. The green profiles correspond to experiment; red profiles are Gaussian fits.

At first, the X-ray beam spatial profile was characterized only with the upstream lens unit CRL1. The diffraction-limited monochromatic X-ray beam size with the CRL1 unit is ∼150–250 µm at interaction chamber 1 (IC1) (Zastrau *et al.*, 2021 ▸). In our experiment, the beam was directly focused to IC1. In Fig. 2 ▸, an image of the beam measured at a photon energy of 9 keV is presented. It can be clearly seen that the beam size is consistent with theoretical considerations; however, the intensity profiles taken across vertical and horizontal directions has a small astigmatism with ratio FWHM_horiz_/FWHM_vert_ = 160 µm/190 µm = 0.84 and the base of the intensity profile in the vertical direction is broader compared with the Gaussian one. The observed loss of the waist symmetry and spreading of the shape can be caused by mirror clipping due to beam drift and/or slight geometrical imperfections of the lens.

### 3.2. X-ray focus characterization after CRL3

**Figure 3:**
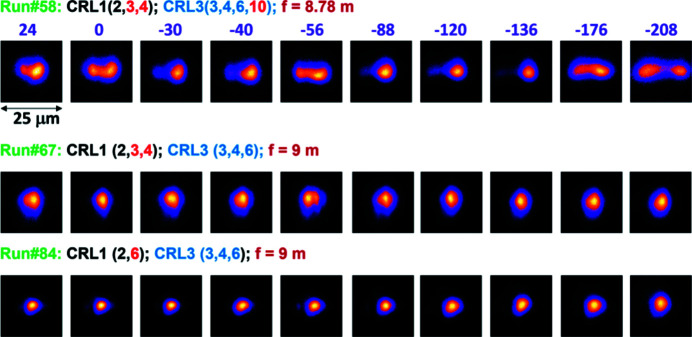
Sequences of LiF images measured along the XFEL beam propagation (with *Z* varied from *Z* = 24 mm to *Z* = −208 mm) for three runs with different configurations of CRL units.

**Figure 4:**
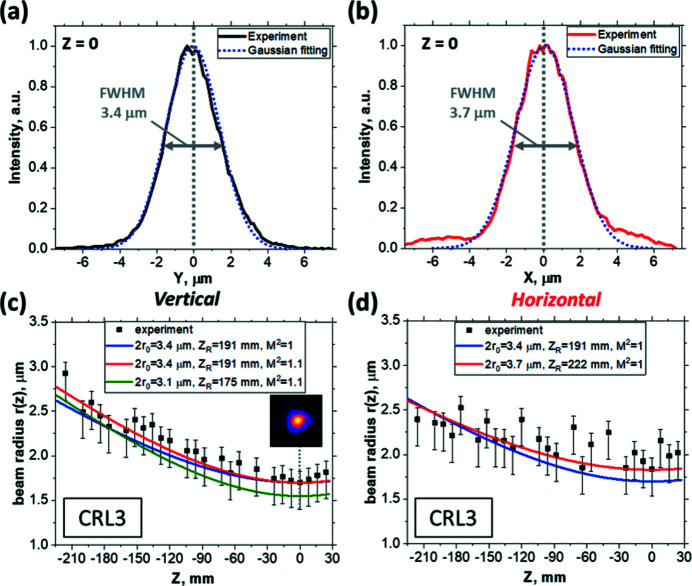
Parameters of the beam obtained in run #84 with combinations of lenses CRL1 (2, 6) and CRL3 (3, 4, 6): intensity profile in the spot with the smallest size (waist at *Z* = 0) in the vertical direction (*a*) and horizontal direction (*b*); dependence of beam radius *r*_*z*_ (FWHM = 2*r*_*z*_) on position *Z* in the vertical plane (*c*) and horizontal plane (*d*). Experimentally measured data (black squares) are compared with theoretical caustics (colored lines).

To characterize the intensity distribution of the focused beam, the caustic was imaged in different planes along the beam propagation for different focusing conditions. The expected focal point was at *Z* = 0 mm. Fig. 3 ▸ shows sequences of LiF images recorded in a *Z* range of 240 mm for different sets of CRL1 and CRL3 units. In run #58 we clearly observe a double structure of the X-ray beam that diverges horizontally over the entire *Z* range. This indicates that single elements in the stack CRL3 were most likely misaligned or damaged. Indeed, by consequentially removing the elements one by one from the CRL3 stack, we found that the CRL3 unit provides a much better beam profile without the last lens cartridge (run #67). The beam has less astigmatism in comparison with run #58 and the double beam structure is gone. It is suspected that there may be some damage on the last element. The best intensity distribution in the beam was obtained in run #84 by the additional change of elements in CRL1 (see Fig. 3 ▸). At best the beam diameter in the focal plane (*Z* = 0 mm) was found to be 3.4 µm and 3.7 µm at the FWHM signal level in the vertical and horizontal directions, respectively [Figs. 4 ▸(*a*) and 4(*b*)]. By applying the PL response function to the LiF image obtained in the focal plane, it was found that about 50% of the energy is contained at a FWHM that agrees with the Gaussian intensity distribution inside the beam.

In addition, a comparison of the experimental beam caustic near the focal plane with the calculated one is presented in Figs. 4 ▸(*c*) and 4(*d*). The black dots show the measured spot sizes. Positive parts of the error bars correspond to the statistical error in determining the size of the spot which does not have a perfectly round shape. It should be emphasized that the measured distribution in the LiF images may be blurred by the secondary photoelectron cloud. In this case, the actual spot diameter is smaller than obtained as discussed in Section 2. Therefore, we introduce an error that determines the order of magnitude of the measurement uncertainty of the beam size in the negative part. Our estimates for the radius of the photoelectron cloud come from both theoretical estimates (Grum-Grzhimailo *et al.*, 2017 ▸) and experimental measurements, which showed that the magnitude of this value does not exceed 300 nm. This value was taken into account by increasing a negative part of the error bars for the experimental points in Figs. 4 ▸(*c*) and 4(*d*). We found that the best fit of the experimental points in the vertical plane occurs in the case of *Z*_R_ = 175 mm and *M* ^2^ = 1.1 [olive solid curve in Fig. 4 ▸(*c*)]. From this point we can conclude that the real beam profile is not ideally Gaussian while the quality factor *M* ^2^ is not significantly different to 1. However, as can be seen in Fig. 4(*d*), the caustic in the horizontal plane does not look like a hyperbola and is evidently different from an ideal Gaussian. This most likely originates due to the lens errors, and possible clipping of the beam.

### 3.3. X-ray sub-micrometer focus characterization after CRL4

For some experimental applications the X-ray beam should be focused down to several tens of nanometers. To achieve such a tightly focused beam, the HED instrument is equipped with the CRL4 unit consisting of a stack of short focal length units (focal distances in the range 100–1000 mm are available), contributed by the Helmholtz International Beamline for Extreme Fields (HIBEF) user consortium (https://www.hibef.eu). The CRL4 unit is installed inside the IC1 experimental chamber due to the short focal distance. In this work, we used a CRL4 stack that provided a focal length of ∼300 mm at a photon energy of 9 keV. The expected size of the waist in the focal plane is of the order of 200 nm. This also involves using a custom-produced phase-correction plate. Details of the phase-correction plate are given by Seiboth *et al.* (2017 ▸).

**Figure 5:**
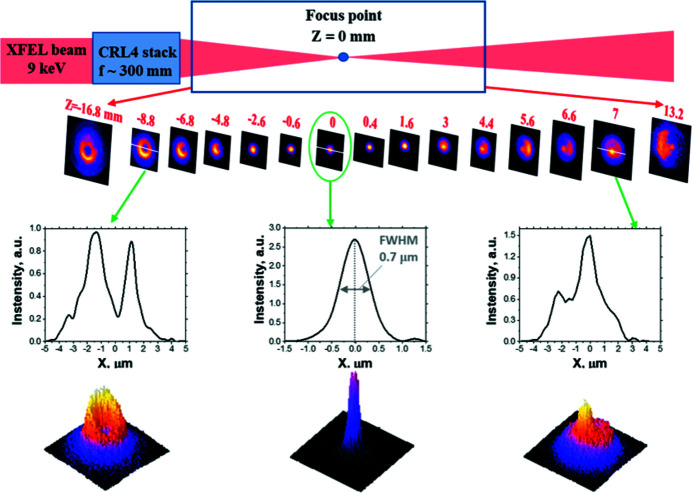
Schematic drawing of intensity distribution measurements of the XFEL beam near the focus position, and sequence of PL images, obtained in one XFEL shot on the surface of the LiF detector in different planes. Lower images are intensity profiles for *Z*-positions of −8.8 mm, 0 mm and 7 mm.

Fig. 5 ▸ shows the intensity distribution of the focused X-ray beam measured by the LiF detector at sequences of planes near the expected focal point. As can be seen in the PL images, the X-ray beam is focused to the point *Z* = 0 mm and then diverges. Due to high sensitivity and large dynamic range of the LiF detector, it was possible without attenuation to measure both the intensity profile of the entire beam far out of focus and in the waist region, where the signal increases by several orders of magnitude. We want to draw attention to the intensity distribution within the cross sections of the focused beam. An interesting evolution of the beam spatial profile was observed along the beam propagation. It is seen in Fig. 5 ▸ that a dark spot in the central part of the beam intensity distribution upstream of the focal position (*Z* < 0) is transformed to a hot spot spike in the same area after the focus position (*Z* > 0). This indicates that Be lenses as the elements of the CRL4 stack have geometrical imperfection. Such structure of the beam distribution can appear due to deviations of the Be lens refractive surface from the ideal parabolic shape as discussed by Zverev *et al.* (2017 ▸) and Celestre *et al.* (2020 ▸).

**Figure 6:**
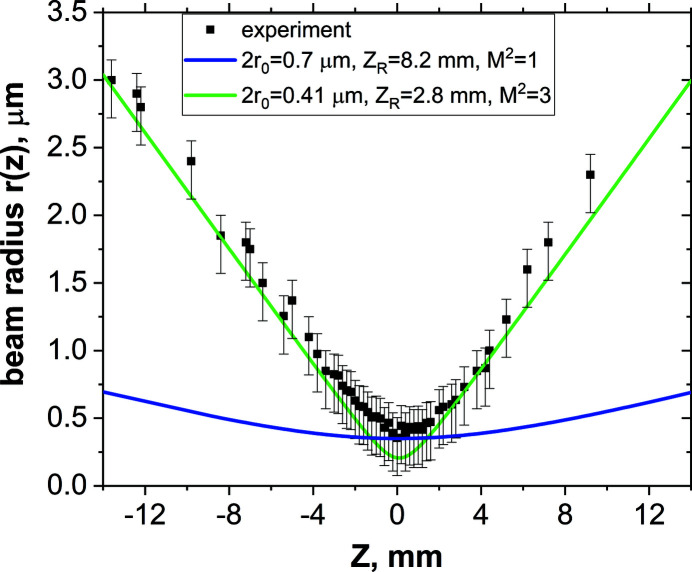
Comparison of the experimental radius *r*(*z*) for set CRL4 measured on the LiF images with the caustic calculated for different parameters *r*_0_ and *M* ^2^.

The minimum beam diameter of 700 nm was measured at distance *Z* = 0 mm (see Fig. 5 ▸). This value is significantly larger than the theoretical value. As discussed in the previous section, we assume that the main reason for that discrepancy is the influence of the secondary electron cascade. To estimate the real beam diameter in the focal plane, we compared a Gaussian profile which fits the shape of experimental caustics in its wings observed out of the focal region (Fig. 6 ▸). The analysis of the caustics was done using equation (1). In Fig. 6 ▸ the dependence of the beam radius *r*_*z*_, measured in the LiF images (Fig. 5 ▸), at position *Z* along the X-ray propagation axis is shown by black squares. In calculations, *r*_0_ and the beam quality factor *M* ^2^ were varied. Considering the smallest beam size measured on the LiF image, a caustic of the Gaussian beam was calculated for parameters *r*_0_ = 0.35 µm and *M* ^2^ = 1. As can be seen in Fig. 6 ▸, in this case, the corresponding curve (in blue) lies completely out of experimental data, with the exception of the area ±2 mm from the focal point. However, by taking into account the error bars, the beam size in the focal plane can correspond to a value of 2*r*_0_ = 0.1 µm. We calculated the beam divergence on the assumption that the focal beam radius *r*_0_ was in the range 0.05–0.35 µm and the beam quality factor *M* ^2^ = 1–4. We found that the best fit of the experimental results takes place in the case of 2*r*_0_ = 0.41 µm and *M* ^2^ = 3 (green solid line). Thus, we may assume that the real focal beam diameter at the FWHM signal level is about 0.41 µm.

The value of the focal spot determined in our experiment exceeds almost twice the value of ∼200 nm that the CRL4 unit is expected to satisfy according to the technical specifications. On the one hand, this may be due to the fact that the spatial resolution of the LiF is not sufficient to accurately determine the size of the beam in the focal plane; however, the beam caustics at distant points in Fig. 6 ▸ show that the size was still larger than 200 nm. Thus, the larger beam size is most likely related to lens aberrations and beam clipping. To test how lensing errors can affect the intensity distribution within a focused beam, we simulated the propagation of X-rays through the CRL4 stack as part of our study.

All simulations were made using the browser-based GUI framework *Sirepo* by RadiaSoft (Rakitin *et al.*, 2018 ▸). It allows running beamline simulations on a personal computer via *Synchrotron Radiation Workshop* (*SRW*) code (Chubar & Elleaume, 1998 ▸). The capabilities of the *SRW* code for X-ray lens modeling have been presented, for example, by Celestre *et al.* (2020 ▸).

**Figure 7:**
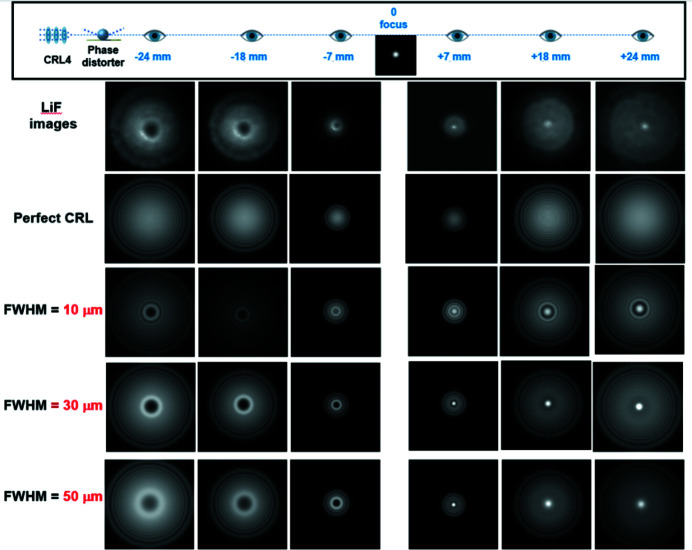
Influence of CRL4 shape imperfection on intensity distribution in the focused XFEL beam. Comparison of experimental observation (upper row of images) with simulations performed for different degrees of parabolic shape imperfection.

Schematically the optical elements used in the simulated beamline and watchpoints of the simulated images are shown in the upper part in Figs. 7 ▸ and 8 ▸. In our simulation, the Gaussian X-ray beam with a photon energy of 9 keV passes through the Be lens stack (refractive index decrement 4.20757 × 10^−6^, attenuation length 7.31 mm) which corresponds to the CRL4 unit used in our experiment with the following parameters: number of lenses *N* = 20, radius of parabolic curvature *R* = 50 µm, web thickness *D* = 30 µm, and aperture size = 316 µm (see Table 1 ▸). For this case, the focal length of the lens stack is 303 mm and the diffraction-limited spot size is ∼200 nm. The beam intensity distribution was obtained in the distance range ±24 mm from the focal plane.

To reproduce manufacturing errors in the CRL4 stack and estimate a shape and phase imperfection, a thin beryllium slice (used as phase distorter) was placed directly behind the lens casing along the optical path in the simulated beamline. We consider commonly encountered fabrication errors in the optical imperfections in refractive lenses such as deviation of shape from parabolic as well as error in the wall thickness at the tip of the parabola. The thickness profile of the phase distorter from beryllium was set as the Gaussian shape distribution. This simple model allows the deviation of the lens from the ideal parabolic surface to be taken into account. We were able to simulate the effects of figure errors on beam shape and intensity along the optical axis. The width (FWHM) of the phase distorter determined the deviation of the shape lenses from the ideal parabolic, and the thickness (*D*) specified the total error in the wall thickness at the tip of the parabola along the beam propagation path.

At first, the *SRW* simulations were performed for X-ray beam propagation through an ideal CRL4 stack (without phase distorter). The image series ‘Perfect CRL’ for this case is shown in Fig. 7 ▸. As can be seen, the modeled images differ from the experimental ones, in which aberration is observed. This is due to the imperfection of the parabolic shape for the refracting surface of CRL4. As the next step, a phase distorter after the CRL4 stack was used in the simulation. At first, the FWHM of the phase distorter only was varied from 10 µm up to the radius of curvature of the CRL4 lens, 50 µm. As seen from Fig. 7 ▸, the best fit is observed for the FWHM of 30 µm.

**Figure 8:**
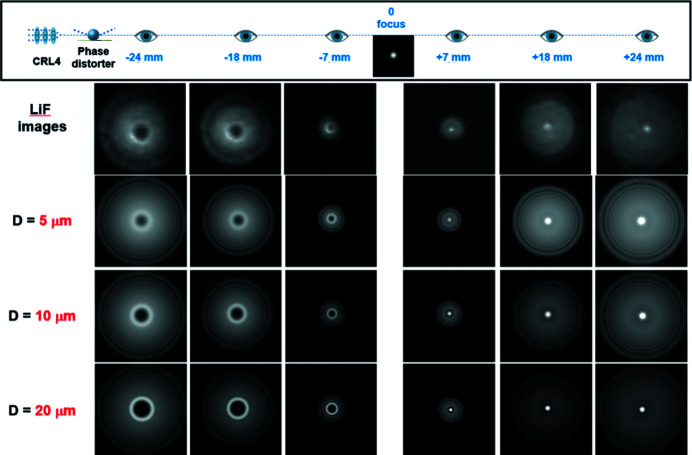
Same as in Fig. 7 ▸ for modeling with a change in the thickness of the phase distorter in the range *D* = 5–20 µm (parameter FWHM = 30 µm fixed).

Fig. 8 ▸ shows the simulation results for when varying the thickness *D* of the phase distorter in the range 5–20 µm. Experimentally recorded images of shaped beams are in good agreement with computer calculations. Performed simulations for the phase distorter with parameters FWHM = 30 µm and *D* = 10 µm are in good agreement with experimental data. However, it is clearly seen that the experimentally observed beam distribution is far from the expected one for perfect CRL4. The value *D* = 10 µm corresponds to an imperfection in the total wall thickness in the CRL stack of less than 1%. These results confirm that the output intensity distribution of the focused beam is extremely sensitive to the quality of the manufacturing and precision of assembling the CRL elements.

## 4. Conclusion

The capabilities of compound refractive focusing systems available at the HED instrument of the European XFEL facility were studied by direct imaging methods applying LiF fluorescent detection. Initial impressions of the focusing capabilities and qualities of the CRL3 and CRL4 units were recorded in the interaction chamber 1. Focus profiles were interpolated to sub-micrometer precision based on recording of the transitional focus of the CRL along it focusing axis. The images also revealed a micrometer-precision intensity distribution of the X-ray profile, indicating various contributions from the beamline optics to the X-ray profiles. The X-ray sensitivity and dynamic range available with LiF crystals would benefit any SASE-based X-ray facility, especially for beamlines with scientific scopes that are greatly dependent on the X-ray pulse profile and focus qualities.

## Figures and Tables

**Table 1:** Beryllium compound refractive lenses used in the experiment on diagnostics of the X-ray focusing at the HED instrument (Zastrau *et al.*, 2021 ▸)

	Number of lenses	Radius of curvature, *R* (mm)	Web thickness, *D* (mm)	Aperture size (mm)
CRL1	3	4.0–5.8	0.03–0.05	3.16–3.80
CRL3	24–26	1.0–5.8	0.03–0.05	1.95–3.80
CRL4	20	0.05	0.03	0.316
